# Augmenting Antitumor Immune Responses with Epigenetic Modifying Agents

**DOI:** 10.3389/fimmu.2015.00029

**Published:** 2015-02-04

**Authors:** Erika Héninger, Timothy E. G. Krueger, Joshua M. Lang

**Affiliations:** ^1^University of Wisconsin Carbone Cancer Center, Madison, WI, USA; ^2^Department of Medicine, University of Wisconsin, Madison, WI, USA

**Keywords:** epigenetics, tumor immunotherapy, histone acetylation, antigen presentation, methylation

## Abstract

Epigenetic silencing of immune-related genes is a striking feature of the cancer genome that occurs in the process of tumorigenesis. This phenomena impacts antigen processing and antigen presentation by tumor cells and facilitates evasion of immunosurveillance. Further modulation of the tumor microenvironment by altered expression of immunosuppressive cytokines impairs antigen-presenting cells and cytolytic T-cell function. The potential reversal of immunosuppression by epigenetic modulation is therefore a promising and versatile therapeutic approach to reinstate endogenous immune recognition and tumor lysis. Pre-clinical studies have identified multiple elements of the immune system that can be modulated by epigenetic mechanisms and result in improved antigen presentation, effector T-cell function, and breakdown of suppressor mechanisms. Recent clinical studies are utilizing epigenetic therapies prior to, or in combination with, immune therapies to improve clinical outcomes.

## Introduction

**Figure 1:**
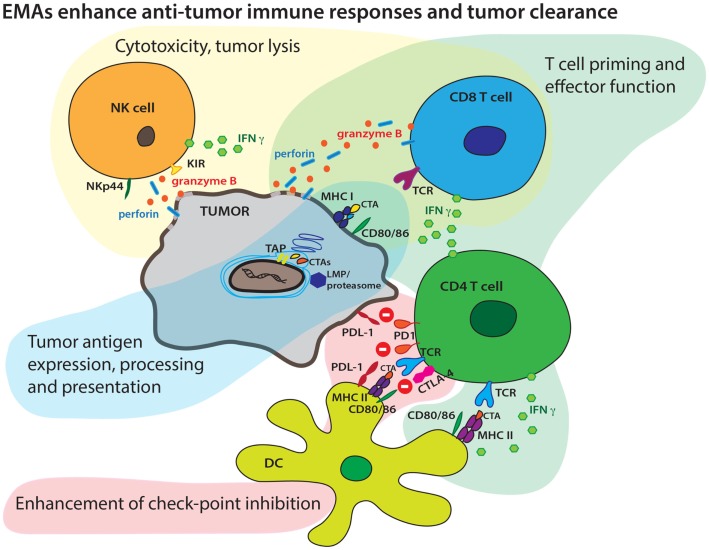
**Epigenetic modifying agents can enhance multiple aspects of an antitumor immune response**. EMAs may boost tumor antigen expression, endogenous antigen processing, increase surface CTA display in context of MHC molecules, and boost presentation to T cells by increasing expression of co-stimulatory molecules. EMAs may also enhance both cellular and cytokine-mediated effector T-cell mechanisms and tumor lysis. EMAs may alter checkpoint inhibition targeting the PD1/PD-L1 and CTLA-4/CD28 axis resulting in more efficient effector T-cell mechanisms.

Immune evasion is a complex phenomenon that entails alterations in cancer cells and the microenvironment to inhibit recognition of tumor cells by immune infiltrating cells. This process includes altered expression and presentation of tumor-associated antigens (TAAs) and secretion of cytokines that promote a regulatory/inhibitory milieu of antigen-presenting cells (APC) and cytolytic T cells (CTL). This complex process is driven by a multitude of factors including altered epigenetic marks in tumor cells that control gene expression. A growing body of literature also suggests that epigenetic alterations can alter immune cell phenotype and function, for both regulatory and cytolytic function. These epigenetic modifications include alterations in DNA methylation and histone modifications, such as acetylation and methylation. The accumulation of epigenetic alterations during tumorigenesis contributes to profound changes in genome-wide transcriptional regulation and genetic stability that promotes immune evasion. The availability of state-of-the-art technologies to screen epigenetic alterations across a variety of malignancies has advanced our understanding of defects in tumor regulation, and new therapeutic approaches have been devised and studied to reverse epigenetic silencing. Since site-specific epigenomic patterns may associate with disease progression, epigenetics has also come into focus for biomarker research (1). These reciprocal fields identify potential therapeutic strategies with integrated biomarkers focused on epigenetic mechanisms to improve antitumor immune responses (Figure 1).

## Introduction to Epigenetics

### Methylation and hypomethylating agents

DNA methylation is one of the most studied epigenetic phenomenon and involves the enzymatic conversion of cytosine residues to 5-methylcytosine. This reaction is catalyzed by the five known mammalian DNA methyltransferases (DNMTs) DNMT1, 3A, 3A2, 3B, and 3L (2). The conversion is typically restricted to cytosine residues of cytosine-guanine dinucleotides called CpG sites. CpG islands contain a high density of CpG sites, which are mostly unmethylated in normal tissues (3). However, most cancers are characterized by localized aberrant DNA hypermethylation of CpG islands within the promoters of various genes (4), including tumor suppressors involved in cell cycle control, cell growth, apoptosis, cell adhesion, DNA repair, angiogenesis, and cell adhesion (5). Furthermore, DNA hypermethylation inhibits gene expression, as evidenced by numerous studies correlating promoter methylation with transcriptional repression during both normal processes as well as tumorigenesis (6–8).

Hypomethylating agents can be used to counteract hypermethylation and restore gene expression. Hypomethylating agents can generally be grouped into (a) nucleosidic or (b) non-nucleosidic DNA methylation inhibitors (Table 1).

**Table 1:** **Hypomethylating agents currently in development**.

Drug	Drug description	Development stage	Reference
**CYTOSINE ANALOGS (COVALENTLY BIND dNMTs)**			
Azacytidine	Among the best-studied agents, but has several disadvantages including high toxicity and a short half-life. Can also incorporate into RNA, reducing specificity	FDA approved	(9)
Decitabine	Decitabine has a longer half-life than azacytidine and only incorporates into DNA, increasing specificity over azacytidine	FDA approved	(10)
SGI-110	A dinucleotide of decitabine and deoxyguanosine that results in a high resistance to cytidine-deaminase cleavage. This may increase exposure time to decitabine while increasing metabolic stability	Phase II	(11)
Zebularine	Lacks the 4′-amino group but covalently traps DNMTs similarly to other analogs. This drug has less toxicity and increased chemical stability when compared to azacytidine or decitabine	Pre-clinical	(12)
CP-4200	An azacytidine derivative with an elaidic acid bound to the molecule that permits diffusion through the cell membrane independent of common nucleoside transporters, thus increasing cellular uptake but possibly decreasing specificity	Pre-clinical	(13)
**ADENOSINE ANALOGS (INCREASE COMPETITION FOR DNMT SAM-BINDING SITES)**			
Cladribine	Inhibits SAH hydrolase, which increases SAH concentration, thus increasing competition for the SAM-binding sites of DNMTs	FDA approved	(14)
Fludarabine	Similar to cladribine	FDA approved	(14)
Clofarabine	A hybrid of fludarabine and cladribine	FDA approved	(15)
**NON-NUCLEOSIDE INHIBITORS (BLOCK DNMT1 ACTIVE SITE/CATALYTIC ACTIVITY**)			
Procainamide	Reduces DNMT1’s binding affinity to SAM and methylated DNA. Binds DNA sequences with high CpG density, blocking DNMT translocation	FDA approved	(16)
Procaine	Inhibits DNMT by masking enzyme target sequences	FDA approved	(17)
Hydralazine	Exact mechanism is controversial. Is thought to bind and block the DNMT1 binding site	Phase III	(16)
Disulfiram	Hypothesized to inhibit the catalytic cysteine of DNMT1	Phase III	(18)
RG108	A small molecule inhibitor of DNMT1 that acts by binding its active site with no detectable toxicity	Pre-clinical	(19)
IM25	A procainamide derivative and small molecule inhibitor of DNMT1. It has been shown to be as potent as but less toxic than azacytidine	Pre-clinical	(20)
Nanaomycin A	A small molecule inhibitor of DNMT1 that is selective for DNMT3B, which is known to be critical for *de novo* methylation	Pre-clinical	(21)
**NATURAL COMPOUNDS**			
Genistein	Decreases DNMT1, DNMT3A, and DNMT3B concentration in prostate cancer cells, but the extent of altered DNA methylation is unclear	Phase III	(22)
Equol	Isolated from soy beans, equol has been shown to have some hypomethylating effect; however, its role in cancer is controversial, and it may even increase the viability of metastatic cancer cells	Phase III	(23)
Curcumin	Binds DNMT1 and blocks its catalytic function with potency similar to some synthetic, non-nucleoside DNMT inhibitors	Phase III	(24)
EGCG	A component of green tea that is shown to have chemopreventive characteristics. Functions as a DNMT inhibitor by depleting the amount of SAM available, leading to decreased DNMT activity	Phase III	(25)
Resveratrol	Found in grapes, resveratrol may function by blocking acetylation of STAT3 and preventing STAT3-mediated targeting of DNMT1 to promoter CpG islands	Phase II	(26)
Parthenolide	Binds the catalytic cysteine of DNMT1 with low potency	Pre-clinical	(27)

Nucleosidic DNA methylation inhibitors are incorporated into the genome during DNA replication. Thus, this class of agents acts only in tumor cells actively undergoing cell division. Agents such as Azacitidine (AZA) and 5-aza-2′-deoxycitidine (5AZA2) were originally synthetized in the 1960s to use as cytotoxic drugs with potential anti-leukemic activity (9, 28, 29). However, their effect on DNA methylation was not identified until later in the process of drug development. 5AZA2 incorporates into DNA in place of cytidine during S-phase and covalently binds DNMTs during the process of DNA replication to ultimately prevent DNA methylation. 5AZA2 has a dual, dose-dependent antineoplastic action. At high doses, it covalently traps DNMT into DNA leading to cytotoxicity. At lower doses, it suppresses tumor growth primarily via hypomethylation of promoter CpG islands of tumor-suppressor specific loci (9, 30). AZA is similar to 5AZA2 but can also incorporate into RNA in the form of azacytidine-triphosphate and directly inhibit protein synthesis.

The restoration of gene expression mediated by hypomethylating agents can impact tumor growth in a wide variety of mechanisms. In prostate cancer (PC), 5AZA2 targets multiple genes including the tumor-suppressor miR-146a microRNA and the androgen receptor (AR). 5AZA2-induced miR-146a induction correlated with both delayed tumor growth and disease progression of castrate-resistant PC (CRPC) in an LNCap xenograft model. The miR46a promoter methylation pattern was also suggested as a biomarker for progression from androgen-dependent to androgen-independent phases of PC (1). Hypermethylation of the AR promoter was shown to associate with PC tumorigenicity and the therapeutic potential of epigenetic agents in addition to anti-androgen therapy has been suggested in several pre-clinical studies both *in vitro* and *in vivo*. 5AZA2 reduced tumorigenicity and cell proliferation of PC cell lines and PC stem/progenitor cells via AR promoter demethylation and AR induction (31). 5AZA2 also restored the antiproliferative and pro-apoptotic effects of the AR-antagonist bicalutamide (BCLT) in both *in vitro* and *in vivo* xenograft models (32). A second-generation derivative, 5AZA2-*p*-deoxyguanosine (SGI-110) was formulated to protect 5AZA2 from cytidine-deaminase inactivation and prolong half-life. SG-110 efficiently retarded tumor growth in an EJ6 bladder cancer xenograft model with less toxicity compared to 5AZA2 *in vivo* (33). Zebularine is a cytidine analog displaying both cytidine-deaminase and DNMT inhibitor properties (34). An *in vitro* study treated breast cancer cell lines with zebularine, potentiating the antitumor effects of other epigenetic drugs including 5AZA2 and SAHA by inhibiting tumor proliferation and clonogenic potential. Other pre-clinical studies in AML and solid tumors found growth inhibition by zebularine via cell cycle arrest and apoptosis induction via various pathways including p53-dependent endoplasmic reticulum (ER) stress (35, 36).

Non-nucleosidic DNA methylation inhibitors directly inhibit DNMT activity without incorporating into nucleic acids. The best-studied agents in this class include hydralazine, procaine, and procainamide. Hydralazine has been studied alone or in combination with valproate acid/magnesium valproate in refractory solid tumors, and it was shown to restore chemosensitivity in gemcitabine-resistant CaLo cervical cancer cell lines via histone methyltransferase inhibition (37, 38). Hydralazine treatment resulted in significant dose- and time-dependent growth inhibition, increased apoptosis, DNA damage, cell cycle arrest, and decreased invasiveness of DU145 PC cells via blockage of the EGF-receptor pathway (39).

Procaine and procainamide are both derivatives of 4-aminobenzoic acid, ester- and amide-, respectively. Procainamide is a competitive inhibitor of DNMT1 hemimethylase activity (40). In an MDA-231 xenograft model, both procainamide and hydralazine demonstrated potent tumor-suppressor reactivation including demethylation and re-expression of the estrogen receptor (41). Procaine suppressed growth of MCF-1 breast cancer cells simultaneously with demethylation events (17). New DNMT inhibitors developed by conjugation of procainamide to L-RG08 or phthalimide showing strong cytotoxicity against DU145 and HCT116 cell lines (42).

Natural plant-derived compounds have been also identified as non-nucleosidic DNA methylation inhibitors and have been extensively studied for global DNA methylation and tumor inhibitory effects. Curcumin was shown to reactivate expression of the Neurog1 gene via promoter CpG site demethylation in LNCap cells. The promoter methylation status of Neurog1 was proposed as a potential biomarker to detect early PC (43). Genistein inhibited the AKT signaling pathway in PC cells via demethylation and deacetylation of histone-H3-lysine 9 at the PTEN, CYLD, p53, and FOXO3 tumor-suppressor gene promoters (44). Resveratrol was also shown to demethylate several tumor-suppressor promoters and restore estrogen sensitivity in triple-negative breast cancer cells by increasing estrogen receptor expression via the inhibition of STAT3 acetylation (26). A clinical trial has been initiated to analyze tissue gene expression patterns in PC patients after genistein treatment (NCT01126879).

### Histone acetylation and histone deacetylase inhibitors

Histone modifications can increase spatial separation of DNA from protein in the nucleosome to permit transcription factor binding to promoter regions, leading to enhanced global gene expression. Chromatin acetylation is regulated by the balanced action between histone acetyltransferases (HAT) and deacetylases (HDAC). Histone deacetylase inhibitors (HDIs) block the catalytic domain of HDACs preventing chromatin condensation and transcriptional repression. HDIs have been intensively studied as potential anticancer compounds both alone and in combination with other therapies in a wide variety of solid tumors and hematologic malignancies with strong pre-clinical evidence for antitumor activity (Table 2). In addition to increasing gene expression of various tumor suppressors, HDIs have also been shown to exert tumor-selective apoptosis induction and proliferation arrest via multiple mechanisms including induction of p21 and the TRAIL-pathway (45). One HDI, suberoylanilide hydroxamic acid (SAHA), suppressed tumor growth, invasion, and migration of highly aggressive ovarian carcinomas by modulating a variety of phenotype-related molecules including members of the caspase pathway, the cell cycle regulator Cyclin B1, tumor-suppressor genes (p21, p53), and tissue remodeling MMP-9 enzyme, among others (46). SAHA promoted autophagy both alone and in combination with 5AZA2 (47, 48) and enhanced T-cell and NK cell-mediated tumor cell targeting by re-sensitizing tumor cells for the TRAIL/Apo2L death receptor pathway in various cancer types (49–54). Panobinostat (or LBH589) displays robust growth inhibition of a wide range of melanoma phenotypes via direct cytotoxicity and cell cycle arrest and is known to induce cell death independently from the apoptotic machinery or death receptor pathways, likely via mitochondrial damage (55, 56). Entinostat has been tested in combination with AZA and SG-110 in lung cancer models, and these combination therapies highlighted robust gene expression changes in key pathways of antitumor mechanisms including cell cycle, apoptosis, tissue remodeling, and DNA damage (57, 58).

**Table 2:** **Histone deacetylase inhibitors**.

Drug	Target	Development	Reference
**HYDROXAMATES**			
Vorinostat	Class I and II HDACs	FDA approved	(59)
Panobinostat	Class I, II, and IV HDACs	Phase III	(60)
Belinostat	Class I and II HDACs	Phase II	(61)
Abexinostat	Class I and II HDACs	Phase II	(62)
Givinostat	Class I and II HDACs	Phase II	(63)
Resminostat	Class I and II HDACs	Phase II	(64)
Quisinostat	Class I and II HDACs	Phase II	(65)
Pracinostat	Class I, II, and IV HDACs	Phase II	(66)
Dacinostat	Class I and II HDACs	Phase I	(67)
Pyroxamide	HDAC1	Phase I	(68)
CHR-3996	Class I HDACs	Phase I	(69)
CBHA	Class I and II HDACs	Pre-clinical	(70)
Trichostatin A	Class I and II HDACs	Pre-clinical	(71)
Oxamflatin	Class I and II HDACs	Pre-clinical	(72)
MC1568	Class IIa HDACs	Pre-clinical	(73)
Tubacin	HDAC6	Pre-clinical	(74)
PCI-30451	HDAC8	Pre-clinical	(75)
**BENZAMIDES**			
Entinostat	Class I HDACs (excluding HDAC8)	Phase III	(76)
Tacedinaline	HDAC1, HDAC2, and HDAC3	Phase III	(77)
Mocetinostat	Class I HDACs	Phase II	(78)
Chidamide	HDACs 1, 2, 3, and 10	Phase II	(79)
BML-210	Class I and II HDACs	Pre-clinical	(80)
M344	Class I and II HDACs	Pre-clinical	(81)
**ALIPHATIC ACIDS**			
Valproic acid	Class I and IIa HDACs	Phase III	(82)
Butyrate	Class I and IIa HDACs	Phase II	(83)
Sodium butyrate	Class I and II HDACs	Pre-clinical	(84)
**CYCLIC PEPTIDES**			
Romidepsin	HDAC1 and HDAC2	FDA approved	(85)
Trapoxin A	Class I and IIa HDACs	Pre-clinical	(86)
Apicidin	Class I HDACs	Pre-clinical	(87)
**SIRT INHIBITORS**			
Nicotinamide	SIRT1	Phase III	(88)
Splitomicin	SIRT1	Pre-clinical	(89)
EX-527	SIRT1	Pre-clinical	(90)
Dihydrocoumarin	SIRT1	Pre-clinical	(91)
Tenovin-D3	SIRT2	Pre-clinical	(92)
AGK2	SIRT2	Pre-clinical	(93)
AEM1 and AEM2	SIRT2	Pre-clinical	(94)
Cambinol	SIRT1 and SIRT2	Pre-clinical	(95)
Sirtinol	SIRT1 and SIRT2	Pre-clinical	(96)
Salermide	SIRT1 and SIRT2	Pre-clinical	(97)
Tenovin-6	SIRT1 and SIRT2	Pre-clinical	(98)
**OTHER**			
TMP-269	Class IIa HDACs	Pre-clinical	(99)
Psammaplin A	Class I HDACs	Pre-clinical	(100)
Nexturastat A	HDAC6	Pre-clinical	(101)
RGFP966	HDAC3	Pre-clinical	(102)

Romidepsin is a unique natural compound originally isolated in the early 1990s from Gram-negative *Chromobacterium violaceum* from a Japanese soil sample and was FDA approved in 2009 for the treatment of cutaneous T-cell lymphoma. Romidepsin was originally described as an anti-Ras molecule and later identified as an HDAC inhibitor. Romidepsin induces G1 phase cell cycle arrest (103) primarily via p21 induction (103, 104).

Sirtuins are potent regulators of cell division that promote survival. SIRT inhibitors have been tested as potential candidates for novel anticancer agents in pre-clinical studies. Sirtinol, a SIRT1/2 inhibitor, suppressed growth of MCF-7 breast cancer cells via various pathways including G1 cell cycle arrest and induction of apoptotic machinery by PARP cleavage, cytochrome c release, Bax up-regulation, and BCL-2 down-regulation. Furthermore, sirtinol elevated autophagy-related markers and increased tumor-suppressor p53 acetylation (105). Inhibition of p53 deacetylation was also shown as a key pathway of tumor suppression and cytotoxicity exerted by two novel SIRT2-specific inhibitors, AEM1 and AEM2 (94).

## Epigenetic Modifying Agents in Cancer Therapy

Clinical utility of epigenetic modifying agents (EMAs) has been demonstrated in a growing body of clinical research studies. Current FDA-approved EMAs include DNMT inhibitors Vidaza (AZA), Dacogen (5AZA2) for AML and MDS, and HDAC inhibitors Istodax (romidepsin) and Zolinza (vorinostat) for treatment of cutaneous T-cell lymphoma. AZA and 5AZA2 have provided a significant advancement in treatment of high-risk hematologic malignancies; however, their clinical efficacy and therapeutic value in solid tumors is limited. This is due to many factors including both the relative molecular instability and significant toxicity of these agents. These dose-limiting toxicities include myelosuppression, fatigue, and infection (106). Furthermore, the lack of clinical benefit at the maximally tolerated doses (MTD) of these agents in patients with solid tumors significantly hindered interest in further clinical development. However, recent studies utilizing EMAs with doses lower than the MTD have found significant antitumor effects renewing interest in these agents. Further pre-clinical work has led to the development of alternative formulations to decrease toxicity and improve pharmacokinetics of these agents (107, 108). For example, the pharmacokinetic profile of the second-generation derivative, 5-aza-2′-deoxycitidine-*p*-deoxyguanosine (SGI-110) is promising, and this agent is being tested in AML, CMML, and MDS in a Phase I/II dose escalation study (NCT 01261312).

Epigenetic aberrations have been shown to associate with resistance to chemotherapy. Several studies addressed the potential of EMAs to reinstate chemosensitivity. When 5AZA2 was administered prior to standard chemotherapy for AML patients, the overall complete remission was 83% (NCT00538876) (109). A recently concluded Phase I/II study on metastatic, docetaxel-resistant CRPC studied the effect of AZA in combination with docetaxel and prednisone and found significant PSA response, favorable clinical outcome, and demethylation of tumor-derived DNA (110). The efficacy of hydralazine and magnesium valproate treatment prior to re-challenge with chemotherapy was addressed in a Phase II clinical trial with patients with refractory, chemoresistant solid tumors (NCT00404508). This study reported a reduction in global DNA methylation, histone deacetylase activity, and 80% of the patients showed clinical benefits with partial response or stable disease (111). A Phase I study in chemoresistant metastatic melanoma (NCT00925132) assessed the potential of sequential epigenetic therapy including 5AZA2 and panobinostat combined with temozolomide chemotherapy. This regimen was generally well tolerated by the cohort with no patient reaching dose-limiting toxicity (112) and has advanced to Phase II testing to further evaluate if EMAs may modify chemosensitivity and apoptosis. A Phase II clinical study of hydralazine and valproic acid in combination with neoadjuvant cytotoxic chemotherapy in locally advanced Stage IIB and IIIA breast carcinoma (NCT00395655) reported an overall response of 81% with complete clinical response in 31% of the patients (113), as well as increased efficacy of conventional cytotoxic agents. A significant decrease in global DNA methylation and in HDAC enzymatic activity was also observed. This study is being continued in a randomized ongoing Phase III study to analyze the efficacy of epigenetic cancer therapy. A Phase II evaluation of efficacy of AZA and/or lenalidomide in relapsed/refractory follicular and marginal zone lymphoma (NCT 01121757) is also ongoing.

Other novel EMAs are in development, such as chromosome-remodeling bromodomain inhibitors (114). These agents have promising pre-clinical data with robust, targeted antitumor effects in a broad range of malignancies. Further clinical development, including evaluation of optimal dosing strategies and toxicity evaluations, will be required to evaluate potential combinatorial strategies with other agents. However, great potential exists for these novel agents to be deployed in a range of clinical contexts.

## Epigenetics and Antitumor Immune Responses

During tumorigenesis, epigenetic alterations play a key role in the suppression of immune recognition and immune surveillance to promote immune evasion via alterations in both the tumor and microenvironment. Immunosuppression is a striking feature of the global methylation pattern of the cancer genome, and it is also a common feature that extends across heterogenous cancer phenotypes. The potential for EMAs to reverse these phenomena is an exciting therapeutic approach to reinstate immune recognition and enhance endogenous tumor clearance. Multiple studies have indicated the potential of epigenetic therapies prior to or in combination with immune therapies, which can act through a variety of mechanisms to enhance antitumor immune responses. These include improving immune recognition via expression, processing, and presentation of TAAs in tumor cells and efficient recognition, T-cell activation, and lysis of tumor targets by immune cells. It is within this complexity of an effective antitumor immune response that epigenetic therapies could play multiple roles.

## Novel Tumor-Associated Antigens

Tumor immune evasion is due, in part, to tolerance for self-antigens and reduced expression of neoantigens. In 1943, Gross published a study demonstrating that foreign antigens expressed by tumor cells may induce immune-mediated rejection of syngeneic tumor grafts (115). The identification of these TAAs and their potential to enhance immunogenicity has been well studied in the field of tumor immunology. These novel TAAs include cancer testis antigens (CTAs) or germline antigens as potential candidates for novel vaccine therapies. CTAs are ideal targets as their expression occurs during tumorigenesis with expression confined to the tumor. The otherwise limited expression in normal tissue beyond the blood-testis barrier suggests these TAAs may be highly immunogenic. However, expression of CTAs in medullary thymic epithelial cells was previously reported (116), and further evidence supported the existence of some level of central tolerance against these germline antigens (117). CTA expression has been detected in a wide variety of hematologic and solid tumors types, although expression levels often vary between disease models, and significant heterogeneity can be observed at different tumor loci within the same host or even the same lesion (118, 119). Lung, ovarian, head and neck, bladder, PC, melanoma, and multiple myeloma have been shown to express a considerable amount of CTAs, and clinical trials have been testing CTA-based therapies with promising results in these groups. Genome-wide analysis has described CTAs as a heterogenous group of antigens characterized by three distinct expression profiles: testis-restricted, testis/brain-restricted, and testis-selective (120). Almost half of the discovered CTAs are linked to the X chromosome (CG-X), which are predominantly testis-restricted. Other CTAs located on various autosomes are typically involved in later stages of germ-cell differentiation with lower antigenic potential (121, 122).

Although their function is not completely understood, prior studies have demonstrated that CTAs play a role in orchestrating cell differentiation processes during germline development. CTAs interact with transcriptional factors driving various signaling pathways involved in gametogenesis, maintenance of genomic integrity, mRNA regulation, metabolic activity, meiosis, and sperm motility. Interestingly, most of the known CTAs show redundancy in germline development as shown in small-animal knock-out models. While the role of CTAs in tumor pathogenesis is even less characterized, data suggest that besides regulating transcriptional activity, CTAs also promote tumor development by supporting cell cycle processes and the mitotic machinery including centrosome formation, mitotic spindle assembly, chromosomal alignment, and nuclear envelope breakdown. Additionally, CTAs promote tumor growth by suppressing apoptosis signaling cascades, inducing aberrant gene expression patterns and impairing response to cancer treatment drugs.

Cancer testis antigens have been proposed for use as biomarkers of disease progression in multiple disease models. Microarray screening of both primary and CRPC revealed remarkable differences in CTA expression. The MAGEA/CSAG family was up-regulated in CRPC but not in primary cancer. Interestingly, PAGEA4 was shown as a strong marker of primary tumors and was silenced in CRPC (123). A study on tumor biopsies demonstrated that SSX expression was an exclusive marker of metastatic lesions and was not detected in primary tumor tissue (124). The expression pattern of 30 CTA antigens was evaluated in glioblastoma samples compared to normal brain tissue. Glioblastoma lines co-expressing three to four CTAs were found to be associated with significantly better overall survival (125). A study in patients with PC (126) detected CTA-specific IgG in sera including NY-ESO-1, LAGE-1, NFX-2, and SSX-2. SSX-2 mRNA levels were also significantly elevated in metastatic PC tissue compared to primary tumors or to benign prostate tissue (126). SSX proteins were also up-regulated in MCH class I-deficient germline cells and in various types of advanced cancers with poor prognosis. SSX-2 was most frequently expressed across prostate cell lines, but SSX-1 and SSX-5 were also inducible after 5AZA2 treatment. SSX expression detected by immunohistochemical tissue arrays in patient tumor samples was restricted to metastatic lesions with no expression detected in primary prostate tumors. Cross-reactive immune responses to a dominant HLA-A2 SSX epitope (p103-111) were observed after immunization of A2/DR1 transgenic mice with SSX vaccines (124).

This tumor-specific expression suggests CTAs may be ideal antigens for tumor-targeting vaccines. In fact, the first CTA was discovered by studying a patient with unusually favorable cytolytic immune responses against melanoma. The CD8-restricted antigen was identified as MAGE-A1 (127). The MAGE family was subsequently recognized as a potent target to enhance tumor-specific CTL cell responses (128) and have been extensively studied as a promising candidate for therapeutic approaches. In 1994, Weber et al. found that 40–50% of tissue samples obtained from patients with advanced melanoma were positive for MAGE-1 from both early and metastatic stages while a wide range of normal tissue samples including tumor-infiltrating lymphocytes, peripheral blood from patients with metastatic melanoma, melanocytes, and benign nevus showed no expression of MAGE-1. Re-induction of MAGE-1 expression in non-expressing cell lines led to HLA-A1 restricted antigen presentation and epitope-specific lysis by CTL (129). HLA-A2-restricted T-cell receptors cloned from SSX-2-seropositive melanoma patients showed epitope-specific reactivity, tumor cell recognition, and tetramer binding when engineered into peripheral blood leukocytes (130). In a DNA vaccine study, improved SSX-2 immunogenicity was reported by introducing peptide ligand modifications to increase binding affinity to HLA-A2 molecules. This enhanced both the magnitude and efficiency of the Th1-type antitumor CD8 T-cell responses and also resulted in a more diverse T-cell-derived cytokine profile (124). Such improved overall efficiency and quality of the epitope-specific responses makes this a promising strategy to enhance tumor-targeting CTA vaccination strategies.

A key regulatory mechanism of CTA expression in both normal and tumor tissue is epigenetic modification (131). Epigenetic silencing is also one of the key changes associated with tumorigenesis (132), and hypermethylation in CTA promoter regions has been observed in various cancer types (133–136). Knockdown models of DNMT1 and DNMT3b demonstrated the role of these enzymes in mediating CG-X antigen gene repression and promoter methylation (135). A considerable body of literature on multiple tumor types has demonstrated that EMAs have the potential to increase immunogenicity via the re-expression of numerous CTAs (126, 129, 135, 137–139). In 1994, Weber et al. reported that 5AZA2 treatment induced MAGE-1 antigen expression in non-expressing cell lines, but not in normal blood cells or melanocytes, and led to HLA-A1-restricted, epitope-specific lysis by CTL (129). Similarly, inducibility of SSX-2 gene in PC LNCap and DU145 cell lines was found following 5AZA2 treatment but not in normal prostate epithelium cell line RWPE1 (126). A subsequent study of the same CTA family demonstrated that While SSX-2 was expressed most frequently in PC cell lines, SSX1 and SSX5 expression was also induced after 5AZA2 treatment (124). In a global screening study of human epithelial cell lines, low-dose AZA treatment up-regulated a wide selection of CTAs, including several members of the MAGE, SSX, SPANX, PAGE families, which were induced in all three tumor types analyzed (breast, colorectal, and ovarian) (140). The inhibition of histone lysine methylation enhanced expression of NY-ESO1, MAGE-A1, and MAGE-A3 expression in H841 lung cancer cells and enhanced tumor cell targeting and lysis by MAGE-A3 and NY-ESO1 epitope-specific T cells (136). HDIs Trichostatin A and depsipeptide FR901228 were both shown to synergize with 5AZA2 to activate CG-X antigen expression in various thoracic and colorectal cancer cell lines (135, 141, 142).

Increased expression of CTAs following treatment with EMAs has been identified in several clinical trials. In a Phase II study for patients with multiple myeloma, sequential AZA and a cytotoxicity-enhancer led to a significant increase of MAGEA4, MAGEA6, SPA17, and AKAP4 in bone marrow compared to pre-therapy samples. The treatment also resulted in enhanced CTAGB1 epitope-specific IFNγ response by patient PBMCs tested *ex vivo* in response to monocyte-derived, CTAGB1-pulsed dendritic cells (DCs) (143). NCT01483274 was proposed to test 5AZA2 efficiency in up-regulating CTA expression followed by a donor lymphocyte infusion and an autologous transfer of MAGE-A1, MAGE-A3, and NY-ESO1 peptide-pulsed DCs in patients with AML who had relapsed after an allogeneic stem cell transplant. Study outcomes are specified as tolerance of study treatment, clinical disease response, and assessment of immune responses to vaccine peptides. The same center proposed a Phase I study with the same treatment to treat relapsed high-risk neuroblastoma, Ewing’s sarcoma, osteogenic sarcoma, rhabdomyosarcoma, or synovial sarcoma. Both of these studies are registered but not yet recruiting.

CTAs have been identified as promising candidates for highly selective tumor-targeting by enhancement of endogenous antitumor responses. Conversely, a vaccine clinical trial with antigenic targets from the same MAGE family, namely MAGE-A3/A9/A12 was just recently terminated due to adverse side effects including substantial neurotoxicity with two vaccination induced fatalities with necrotizing white matter tissue damage and CD8^+^CD3^+^ T-cell neuro-infiltration. Follow-up studies revealed a previously unrecognized expression of MAGE A12 in normal brain tissue, which could be the potential target for the vaccine-mediated neuroinflammatory response detected in this trial. This case has alerted for the need of refining CTA characterization to help identify suitable and safe targets for systemic immunization against cancer (144).

## Antigen Processing and Antigen Presentation

Beyond sufficient expression of TAAs, effector antitumor T-cell responses also require the processing and loading of TAAs onto major histocompatibility complex (MHC) I complexes in the context of co-stimulatory molecules. The MHC I antigen-presenting machinery samples designated ubiquitin-tagged endogenous proteins and delivers them to the proteasome complex low molecular mass polypeptide (LMP) 2, LMP7, LMP10 for preprocessing into up to 25-meric peptides. This peptide-pool is then further cleaved by cytosolic aminopeptidases and delivered to the ER via the transporter associated with antigen processing (TAP) complex (TAP1, TAP2) for subsequential trimming by ER aminopeptidase associated with antigen processing (ERAAP) followed by chaperone (calnexin, calreticulin, ERp57, and tapasin)-mediated assembly and loading onto the antigen-MHC complex. After peptide loading, the chaperones are released from the peptide-MHC complex, which is then trafficked to the Golgi via vesicle transport and delivered for surface membrane display (145). Defects of the MHC I antigen presentation system lead to impairment of immune surveillance, which has been linked to both tumorigenesis and poor clinical outcomes (146–150). A tissue microarray analysis on 71 PC patient samples revealed a significant decrease in beta 2 microglobulin (B2M) expression compared to normal surrounding tissue (145). A study on diffuse large B cell lymphoma revealed aberrant B2M protein expression in 75% of the examined biopsies, which was associated with the loss of HLA-I surface expression (151–153).

Epigenetic alterations have been identified as one of the mechanisms underlying deficient antigen presentation in pre-clinical tumor models. Histone acetylation of the TAP1 promoter was proposed as a potential repressor mechanism accounting for TAP1 deficiency in various carcinoma cell lines. The level of acetyl-histone H3 strongly correlated with the level of TAP1 expression and with metastatic features in malignant carcinomas (154). The reversal of tumor antigen presentation impairment and MHC-complex deficiencies may promote tumor killing. EMA has been shown as a potential approach to reverse such defect and enhance tumor immune responses. Li et al. analyzed the global response to low-dose AZA in 63 human epithelial cancer cell lines and found that B2M, HLA-B, HLA-C, CTSS, NSF2, TAP1, and proteasome proteins PMSB8 and PMSB9 were up-regulated in at least three cell lines each (140). Using a high-throughput bioinformatics approach, Kortenhorst et al. identified a comprehensive list of genes and pathways affected by HDI treatment in PC cell lines and showed a significant up-regulation of MHC genes including HLA-Class I molecules and B2M (145). Antigen processing and presentation is also enhanced by AZA treatment via up-regulation of the interferon type I and type II families including interferon-gamma receptor 1 and STAT1 as was shown in an *in vivo* mouse model for HPV-16-associated tumors and in NSCLC tumor cell lines (153, 155).

Dendritic cells play a pivotal role in TAA sampling, processing, and presentation to T cells. Selective DC targeting makes antigen delivery to the draining lymph nodes more efficient. Fusion proteins with high-affinity DC-specific binding components facilitate DC loading and shield antigens from biodegradation allowing for lower vaccine doses. DC targeting has been shown to be a promising novel vaccination strategy that results in enhanced, durable, and overall higher quality immune responses (156). A phase I trial study has been registered (NCT01834248) and is currently recruiting to test immune response to DEC-205/NY-ESO1 fusion protein (CDX-1401) and 5AZA2 in patients with MDS or AML. CDX-1401 is a full length NY-ESO1 protein sequence fused to a monoclonal antibody against DEC-205, a surface marker present on professional APCs to enhance targeted delivery of peptide antigen to the antigen processing machinery and to enhance the efficacy of DC-mediated T-cell induction. NY-ESO1-specific primed T cells are expected to target tumor more efficiently due to an increase in 5AZA2-induced *in situ* NY-ESO1 expression by the cancer cells. In addition to addressing safety, efficacy, tolerability, and vaccine immune responses, the study also aims to assess the molecular epigenetic response to 5AZA2.

## Epigenetics and Adaptive Immune Responses

Epigenetic silencing of immune-related genes is a striking feature of the global methylation pattern of the cancer genome. The impact of epigenetic alterations in tumorigenesis can foster an immunosuppressive tumor microenvironment. These alterations are potentially modifiable with EMAs. A large-scale study on epithelial cancer cell lines treated with low-dose AZA have identified 80 up-regulated gene sets similar across three cancer types analyzed including ovarian, colorectal, and breast cancer and among those there were 15 commonly up-regulated immune gene sets including elements of the interferon signaling, antigen presentation, influenza, and the chemokine and cytokine families. A similar analysis of lung cancer cell lines then also showed a 95% overlap of the gene sets up-regulated by AZA as well as a relative dominance among those by immune-related pathways. The same study also analyzed primary tumor data in public gene expression data bases and found that the above identified AZA-responder immunomodulatory genes showed clustering into a “low” and “high” expression subgroup across all three epithelial cancer types independent of clinical stage or of most tumor subtypes. Similar findings were concluded when looking at NSCLC and melanoma databases. Strikingly, 11 of the 15 AZA-responder immunomodulatory gene clusters were up-regulated in patient biopsies following an 8-week AZA and entinostat combination therapy. These findings suggest that gene expression profiling may help identify patients with immune evasion phenotype as candidates potentially benefiting from epigenetic therapy (140).

Effective T-cell priming requires the antigen presented in the MHC complex in the context of co-stimulatory molecules, which define the phenotype of T-cell responses. Modifying co-stimulatory patterns in the immune synapsis can effectively alter the magnitude of both effector and regulatory T-cell responses (157). Therapeutic targeting of negative or positive co-stimulatory molecules to enhance antitumor immune responses has been tested in both pre-clinical and clinical studies with promising outcomes. Low-dose *in vitro* 5AZA2 treatment of EL4 cells increased co-stimulatory CD80 expression by the tumor cells, which led to increased immunogenicity detected after engraftment into C57BL/6 mice (158). The addition of AZA and entinostat to treatment with checkpoint-inhibitor anti-PD-1/anti-CTLA-4 antibodies led to remarkable tumor regression in a syngeneic mouse model with checkpoint-inhibitor-resistant metastatic cancer (159). A study in human leukemia cell lines demonstrated that AZA treatment increased the expression of immune-checkpoint molecules PD-1, PD-L1, PD-L2, and CTLA-4 (160). AZA increased both transcript and surface expression levels of PD-L1 on a NSCLC cell line (153).

Targeting regulatory immune responses to enhance antitumor immune responses has been addressed in several recent clinical trials. Clinical response and prolonged stabilization were reported in a substantial proportion of patients with diverse tumor types even with treatment-refractory, metastatic types otherwise considered as non-responsive to immunotherapy. Notably, patients with PD-L1 negative tumors showed no response to therapy (161). A follow-up study of three patients for more than 3 years after cessation of anti-PD-1 therapy showed durable, stabile, or re-inducible complete remission (162). Patients showing no up-regulation of checkpoint elements after treatment with combination therapy of AZA and vorinostat had increased survival (160). The analysis of PD-L1 and PD-1 expression patterns within the tumor microenvironment revealed a strong correlation between tumor cell PD-L1 expression and both the magnitude of intratumoral immune cell infiltration and their PD-1 expression. These findings suggested a strong correlation between tumor PD-L1 expression and clinical response (163). Since EMA treatment has a potential to restore PD-L1 expression on tumor cells, combinatorial EMA treatment may expand the group of candidates for PD-L1 checkpoint-inhibitor therapy. A Phase II study in NSCLC (NCT01928576) is currently recruiting to analyze the efficacy of entinostat and/or azacitidine prior to PD-1 blocker nivolumab treatment.

Enhancement of effector immune mechanisms can benefit antitumor interventions and therapeutic approaches to improve clinical outcomes. Combining EMAs provides a novel alternative to improve antitumor immune responses. Importantly, data from immunodeficient animal models have demonstrated that the tumor inhibitory effect of EMAs requires an intact immune system. The antitumor effect of HDI vorinostat and panobinostat treatment was diminished in RAG2γC^-/-^ and IFNγR^-/-^ immunocompromised animals (164, 165). Pre-clinical studies have demonstrated that hypomethylating agents enhance the effector function of both T cells and NK cells. 5AZA2-treated tumor cells induced a higher yield of tumor-infiltrating CD4, CD8 T cells, and NK cells and IFNγ production was also elevated in both T-cell compartments. Mechanistic experiments within the same study identified a CD8-dependent tumor rejection mechanism induced by 5AZA2 (158). 5AZA2 treatment had a biphasic effect on both the phenotype and function of *ex vivo* expanded normal human peripheral NK cells. After treatment, KIR and NKp44 expression were increased while NKG2D decreased. The 5AZA2-induced hypomethylation followed a U-shaped dose-response curve similar to the effects on cytotoxicity (166). Human peripheral NK cells showed increased cytotoxicity when exposed to exosomes isolated from MS-275-treated HEPG2 cells compared to untreated cancer cells. The TAA chaperone HSP70 and the MHC-related MICA, MICB content of the exosomes was elevated by MS-275 treatment suggesting a non-antigen-specific mechanism selective for NK cell activation (167). Combinatorial panobinostat treatment significantly reduced tumor burden and enhanced TH1 cytokine profile and effector function of adoptively transferred tumor-specific T cells in a melanoma model. This treatment also resulted in a dramatic increase in the tumor-infiltrating effector cell to regulatory T-cell ratio (168).

Previous studies have suggested that EMAs can impact T-cell proliferation, differentiation, and function (169–171). Animal studies support the concept that HDIs can promote a cytotoxic antitumor immune response (172) but numerous studies have shown that patients with PRCA have a profound impairment in the function of circulating and tumor-infiltrating T cells (173, 174). Whether EMAs can promote antitumor responses in this immunosuppressive environment in patients with PRCA is unknown. Another challenge to this hypothesis lies in the clinical findings of myelosuppression due to EMA treatment when given at high doses or intervals (175–177). However, low doses of EMAs may promote T-cell-mediated cell lysis (169).

As with any systemic therapy, there are potential toxicities from EMAs that could inhibit antitumor immune responses or complicate patient outcomes. For example, dose-dependent toxicities with EMAs include leukopenia, granulocytopenia, and thrombocytopenia. Altering dose and schedule of these agents can ameliorate some of these toxicities but must be closely monitored. In addition, reactivation of chronic viral infections, including HIV type 1, was reported following treatment with SAHA in an *in vitro* model for latent infection (178). Virus-related illnesses were also observed in a multi-institutional phase II clinical trial of romidepsin, including EBV, HBV, and VZV reactivation (179). Finally, epigenetic modification has been also suggested to regulate immune mechanisms involved in chronic inflammation and autoimmunity. The extent to which these toxicities may impact patient outcomes is unclear and is the subject of ongoing clinical studies.

## Biomarkers for Epigenetic Therapies

Studies have identified a wide variety of impacts that EMAs can have on both the tumor and immune compartments. However, the toxicities associated with these agents when dosed at traditional MTDs will likely mitigate effective immune responses. Thus, optimal biologic dosing strategies may be key to the success of these therapeutic strategies. Integration of biomarkers for the purpose of assessing methylation and histone modification status in tumor and immune compartments as well as expression of relevant immune-related genes, could identify patient-specific dosing strategies targeting immune activation. State-of-the-art tools are available to dissect and measure immune responses quickly and efficiently using multi-parameter analysis platforms and high-throughput technologies. Improving tumor analytics for these purposes, whether through traditional tumor biopsies or alternate assays for tumor cells in circulation, may further identify optimal biologic dosing strategies to modify gene expression. Given the potential of these agents to alter expression of a wide range of genetic targets, incorporation of discovery biomarkers to identify novel targets would have further clinical and experimental utility. For example, large clusters of genes involved in effector immune mechanisms have been identified as loci accumulating genetic aberrations in the cancer genome. Only a limited number of individual genes from these clusters have been tested so far in either pre-clinical or clinical studies in cancer research. Thus, careful integration of biomarkers into these therapeutic strategies will be critical to advance these clinical hypotheses.

## Conclusion

In recent years, the development of combination immunotherapies has branched out as a robust new approach to novel cancer therapeutics. The interplay between tumor and host during tumor pathogenesis is a complex process and epigenetic modifications mark many components of the pathologic changes leading to tumor evasion. Epigenetic therapies have the potential to reverse this process at multiple levels. Immune therapies provide a significant benefit compared to standard cancer therapies by allowing for better accommodation of tumor heterogeneity and the patient’s individual immune repertoire. Epigenetic agents can complement immunotherapies by enhancing many underlying mechanisms of the antitumor immune response (Figure 1). However, the potential benefits of these agents must be balanced by the potential toxicities of these therapies. Biologic dosing strategies are likely the best approach to maximize benefit while limiting toxicity. Integrated biomarkers across tumor and immune compartments may allow determination of biologic doses, though thoughtful approaches to these assays must be considered.

## Conflict of Interest Statement

The authors declare that the research was conducted in the absence of any commercial or financial relationships that could be construed as a potential conflict of interest.
